# Nanoparticle curcumin ameliorates experimental colitis via modulation of gut microbiota and induction of regulatory T cells

**DOI:** 10.1371/journal.pone.0185999

**Published:** 2017-10-06

**Authors:** Masashi Ohno, Atsushi Nishida, Yoshihiko Sugitani, Kyohei Nishino, Osamu Inatomi, Mitsushige Sugimoto, Masahiro Kawahara, Akira Andoh

**Affiliations:** Department of Medicine, Shiga University of Medical Science, Otsu, Japan; University of South Carolina School of Medicine, UNITED STATES

## Abstract

**Background and Aims:**

Curcumin is a hydrophobic polyphenol derived from turmeric, a traditional Indian spice. Curcumin exhibits various biological functions, but its clinical application is limited due to its poor absorbability after oral administration. A newly developed nanoparticle curcumin shows improved absorbability *in vivo*. In this study, we examined the effects of nanoparticle curcumin (named Theracurmin) on experimental colitis in mice.

**Methods:**

BALB/c mice were fed with 3% dextran sulfate sodium (DSS) in water. Mucosal cytokine expression and lymphocyte subpopulation were analyzed by real-time PCR and flow cytometry, respectively. The profile of the gut microbiota was analyzed by real-time PCR.

**Results:**

Treatment with nanoparticle curcumin significantly attenuated body weight loss, disease activity index, histological colitis score and significantly improved mucosal permeability. Immunoblot analysis showed that NF-κB activation in colonic epithelial cells was significantly suppressed by treatment with nanoparticle curcumin. Mucosal mRNA expression of inflammatory mediators was significantly suppressed by treatment with nanoparticle curcumin. Treatment with nanoparticle curcumin increased the abundance of butyrate-producing bacteria and fecal butyrate level. This was accompanied by increased expansion of CD4^+^ Foxp3^+^ regulatory T cells and CD103^+^ CD8α^−^ regulatory dendritic cells in the colonic mucosa.

**Conclusions:**

Treatment with nanoparticle curcumin suppressed the development of DSS-induced colitis potentially via modulation of gut microbial structure. These responses were associated with induction of mucosal immune cells with regulatory properties. Nanoparticle curcumin is one of the promising candidates as a therapeutic option for the treatment of IBD.

## Introduction

Inflammatory bowel diseases (IBD) comprise two major phenotypes, Crohn's disease (CD) and ulcerative colitis (UC). IBDs are relapsing and remitting conditions that afflict millions of people throughout the world. While the etiology of IBDs remains poorly understood, recent studies suggest that excess activation of the mucosal immune system targeting the gut microbiota plays a pivotal role [1–3].

Curcumin (1,7-bis(4-hydroxy-3-methoxyphenyl)-1,6-heptadiene-3,5-dione) is a hydrophobic polyphenol with a characteristic yellow color derived from turmeric, a traditional Indian spice [4–6]. Turmeric is prepared from the root of the perennial herb *Curcuma longa*, a member of the ginger family. Numerous studies have indicated that curcumin possesses a wide variety of biological functions, such as anti-inflammatory, anti-cancer, anti-oxidant, anti-microbial, wound-healing and hypoglycemic activities [5, 6]. These multi-targeted activities of curcumin have been shown to be mediated by the suppression of various cell signaling pathways including NF-κB, STAT3, Nrf2, ROS and COX-2 [5, 6].

The safety and tolerability of curcumin have been confirmed by human clinical trials [6–9], and further clinical applications for the treatment of various inflammatory and malignant disorders are expected [4, 5]. However, the major limitation of its clinical use is associated with low oral bioavailability due to poor absorption from the gut [6, 10]. Curcumin possesses a highly hydrophobic character and is poorly soluble in water [10]. The low oral bioavailability of curcumin is owing to its poor solubility. Previously, a number of studies of delivery systems to improve oral bioavailability of curcumin have been conducted [11]. Among them, the application of nanotechnology for curcumin usage has markedly improved its water solubility and oral bioavailability [10]. The absorption efficacy of nanoparticle curcumin was 30-fold higher than that of curcumin powder in both rats and humans [10]. In rats, maximum plasma concentrations of curcumin after oral administration of 30mg of nanoparticle curcumin and curcumin powder were 764 ng/ml and 13.0 ng/ml, respectively [10]. Clinical application and the usefulness of nanoparticle curcumin have been already reported in some pathological conditions [10, 12–14].

Curcumin has been reported to be effective in inducing and maintaining remission in patients with UC [9, 15], suggesting a potential application of nanoparticle curcumin for the treatment of IBD. However, there are no basic or clinical reports of nanoparticle curcumin in gastrointestinal disorders including IBD. In this study, we examined the effect of nanoparticle curcumin on the development of dextran sulfate sodium (DSS)-induced colitis. To our knowledge, this is the first basic report of nanoparticle curcumin on an experimental model of IBD.

## Materials and methods

### Animals and DSS colitis

BALB/c mice (six to eight-week-old females) were purchased from CLEA Japan Inc. (Tokyo, Japan) and housed under specific pathogen-free conditions. Mice were allowed free access to water and rodent chow (CE-2; CLEA Japan, Inc.). Nanoparticle curcumin (named Theracurmin) was provided by Theravalues Corporation (Tokyo, Japan). Nanoparticle curcumin was mixed with the powder form of a normal rodent diet (containing 0.2% (w/w) nanoparticle curcumin). The administration of nanoparticle curcumin was started 7 days before DSS administration. Experimental colitis was induced by the oral administration of 3% w/v DSS (molecular weight 5000; Wako Pure Chemical Industries, Osaka, Japan) in distilled water. Mice were divided into 4 groups; control group (Control), nanoparticle curcumin group (Theracurmin), DSS group (DSS) and DSS plus nanoparticle curcumin group (DSS+Theracurmin). Mice were euthanized on day 18 under isoflurane anesthesia by quick cervical distortion to minimize animal suffering. Mice were monitored for health and weight daily by experienced keepers, and were euthanized if they presented with a reduced general condition such as erected fur and /or altered social behavior, or had lost more than 20% of their body weight. None of mice in the study died as a result of sever colitis. All experiments were carried out in strict accordance with the recommendations in the Guide for the Care and Use of Laboratory Animals of the National Institutes of Health. This study was approved by the Research Center for Animal Life Science and Use Committee at the Shiga University of Medical Science (Otsu, Japan) (Permit number:2015-4-1).

### Assessment of DSS-induced colitis

Mucosal inflammation was assessed using the disease activity index (DAI) described previously [16]. Histologic evaluations were performed in a blinded fashion using a validated scoring system [7].

### Epithelial permeability assay

Epithelial barrier function was assessed by *in vivo* permeability assay using FITC-labeled dextran according to the method described previously [17]. Briefly, after 4 h fasting mice were orally administrated with FITC-labeled dextran (44 mg/100 g body weight), (MW 4000; FD4, Sigma-Aldrich Co., St Louis, MO). Blood was collected 5 h later via cardiac puncture and was then spun at 1,000 rpm for 20 min to separate serum from whole blood cells. Fluorescence intensity in the serum was determined at 485-nm excitation and 520-nm emission wavelengths. FITC-dextran concentrations were determined using a standard curve generated by serial dilution of FITC-dextran.

### Human colonic epithelial cell line (HT-29)

The human colon epithelial cell line, HT-29, was obtained from the American Type Culture Collection (ATCC, Manassas, VA). These cells were cultured according to the instructions of ATCC.

### Immunocytochemistry for NF-κBp65

For immunofluorescence, cells were grown on a culture slide system (IWAKI, Tokyo, Japan), fixed with paraformaldehyde and reacted with anti-NF-κB p65 antibody. Then, the cells were incubated with fluorescence-labeled secondary antibody. Nuclei were visualized using DAPI (Vector laboratories, Burlingame, CA). A digital confocal laser scanning microscope (Nikon, Tokyo, Japan) was used for analysis. Immunohistochemical analysis was performed according to the method described previously [18]. The used antibody was listed in S1 Table.

### Real-time PCR analysis

Total RNA was extracted using the TRIzol reagent (Invitrogen, Carlsbad, CA). Total RNA was converted to cDNA using Superscript II (Invitrogen). Real-time PCR was performed using the Light Cycler 480 System II (Roche Diagnostics, Basel, Switzerland). The data were normalized versus β-actin for each target molecule, and are expressed as fold-increases relative to the data of the medium alone (no stimulation). The PCR primers used in this study are presented in S2 Table.

Bacterial DNA was extracted from mouse feces using QIAMP DNA stool mini kit (QIAGEN, Hilden, Germany). The abundance of bacteria species was qualified with the Light Cycler 480 (Roche Diagnostics). PCR primers used in this study are shown in S2 Table. PCR products of the different primer sets were ligated into the plasmid vector and transformed into competent high DH5α (Toyobo Co, Ltd., Osaka, Japan). Plasmid DNA was purified with a MagExtractor (Toyobo Co, Ltd.) and used as standards for real-time PCR [19].

### Terminal restriction fragment length polymorphism (T-RFLP) analysis

DNA samples from feces were isolated using the method described previously [20]. The final concentration of DNA sample was adjusted to 10 ng/μl. T-RFLP analysis of the gut microbiota was performed according to the method described previously [20]. The T-RF fragments were divided into 30 operational taxonomic units (OTUs) as described by Nagashima et al [21]. The prediction of bacteria was performed according to the *Bsl*I-digested T-RFLP database [21].

### Extraction of nuclear proteins

The nuclear proteins from tissues were extracted using a CelLytic NuCLEAR Extraction Kit (Sigma-Aldrich Co., St Louis, MO). Extracted nuclear proteins were subjected to immunoblot for NF-κBp65. Signal detection was performed using the enhanced chemiluminescence immunoblot system (GE Healthcare UK Ltd, Little Chalfont, UK). The used antibodies are listed in S1 Table.

### Cell isolation and flow cytometry

Lamina propria mononuclear cells (LPMCs) were isolated according to the method described previously [22]. Flow cytometric analysis was performed according to a previously described method [22]. The used antibodies are listed in S1 Table.

### Measurement of fecal short-chain fatty acids

High-performance liquid chromatography (HPLC) was carried out for the analysis of stool extracts as previously described [23]. HPLC was performed using an Agilent 1120 Compact LC system (Santa Clara, CA) and a COSMOSIL 4.6 X 150mm 5C18-AR-II column (Nacalai Tesque Inc., Kyoto Japan).

### Statistical analysis

BellCurve^®^ for Excel (version 2.11) (SSRI Co., Ltd., Tokyo, Japan) was used for statistical analysis. The statistical significance of the differences was determined by one-way ANOVA with Bonferroni post hoc test. Differences resulting in *P* values less than 0.05 were considered to be statistically significant.

## Results

### Nanoparticle curcumin attenuates the development of DSS-induced colitis

**Fig 1:**
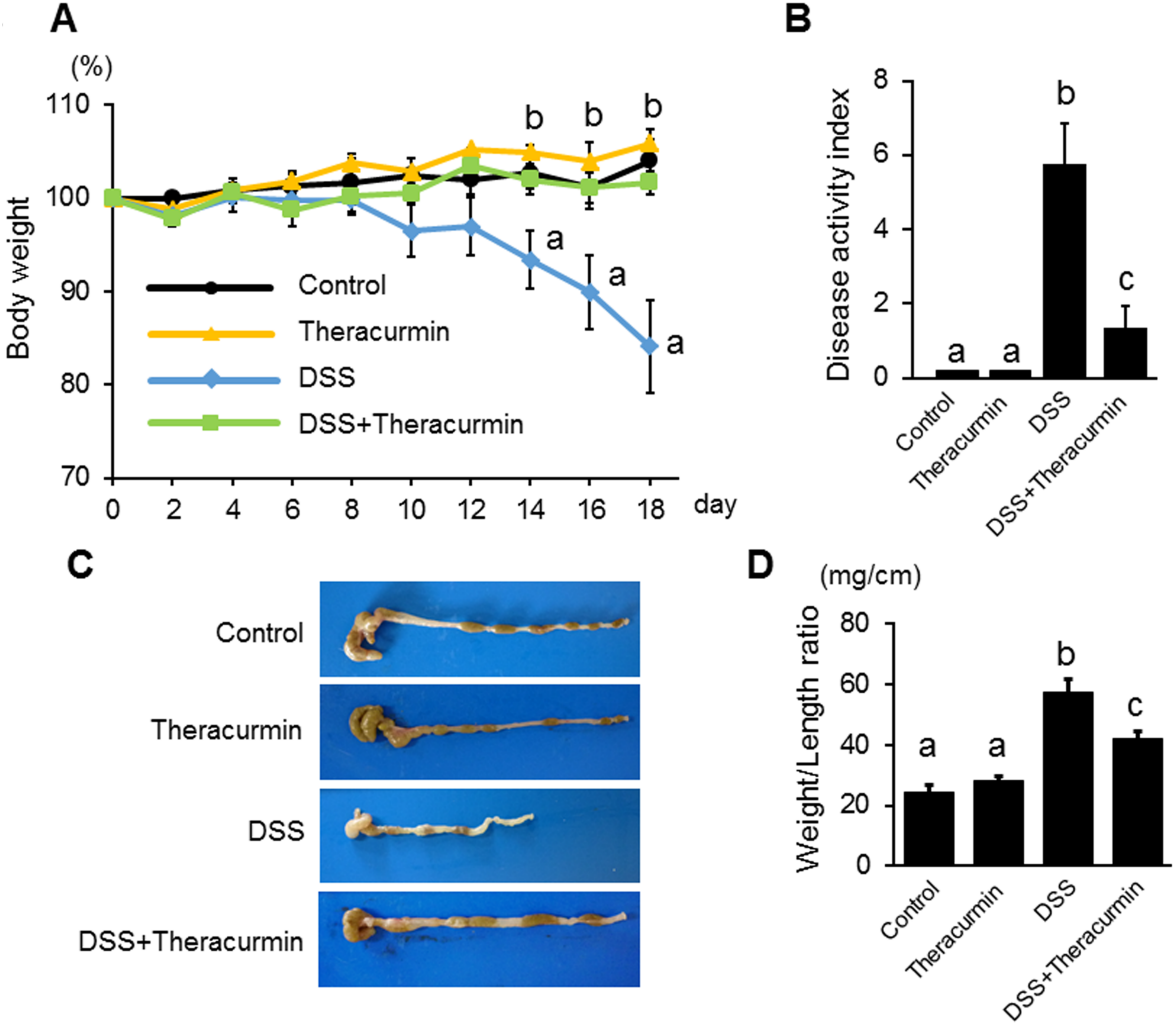
Effect of nanoparticle curcumin on the development of DSS colitis. BALB/cAJcl mice were treated with nanoparticle curcumin (Theracurmin) for 7 days prior to the start of 3% DSS treatment. The mice were sacrificed on day 18. (A) Body weight. (B) Disease activity index. (C) Representative photographs of the colon. (D) Colonic weight/length on day 18. The data are expressed as means ± SEM (n = 6 mice/group). The data are representative of four independent experiments. Values not sharing a letter are significantly different (*P*<0.05).

**Fig 2:**
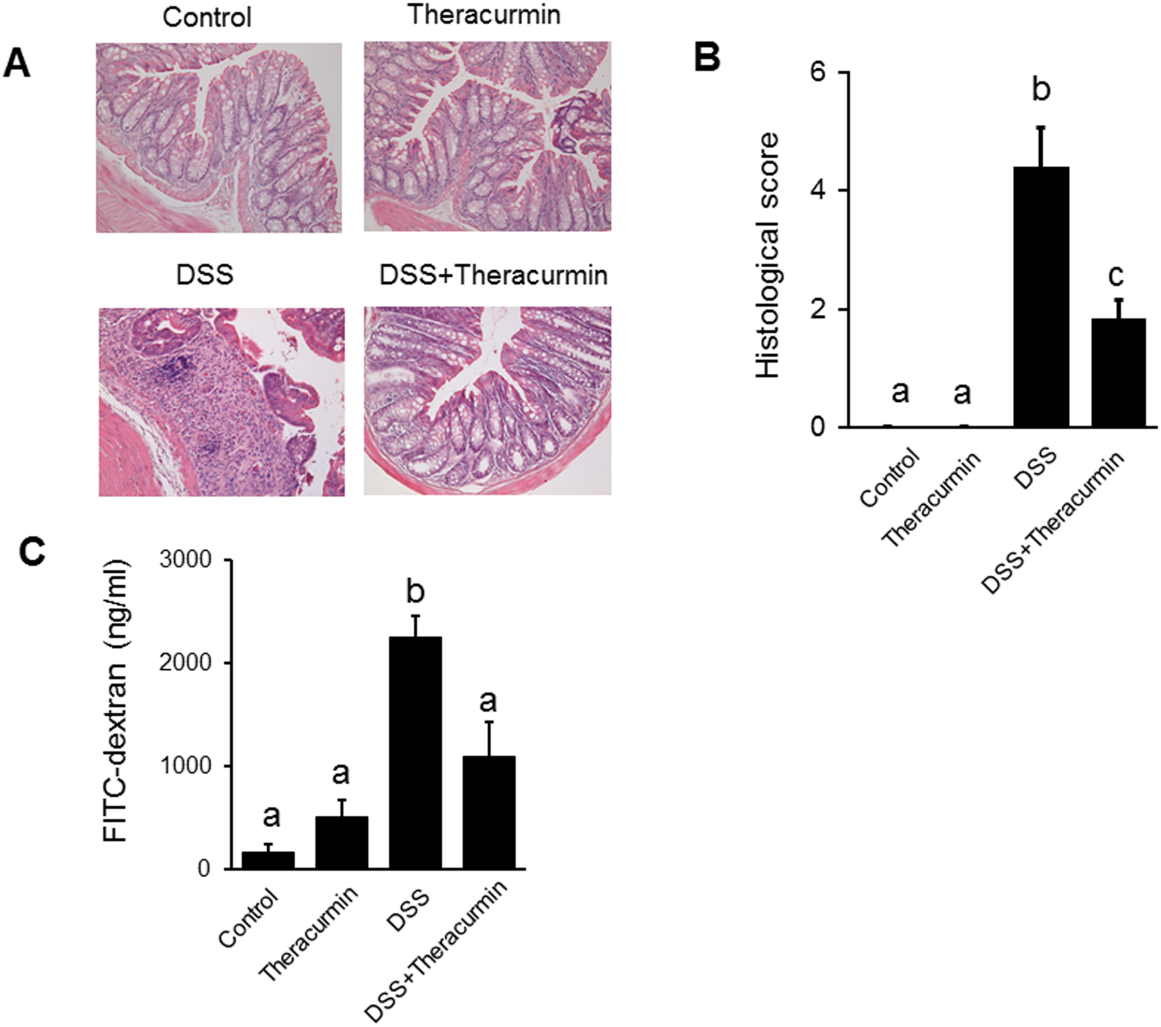
Histological evaluation of colitis. (A) Histological picture of the colonic tissue on day 18. (original magnification ×200.) (B) Histological sore. The data are expressed as means ± SEM (n = 6 mice/group). (C) Epithelial permeability. Mice were orally administrated with FITC-labeled dextran (44 mg/100 g body weight), (MW 4000; FD4, Sigma-Aldrich Co.). Serum was collected 5 h later and fluorescence intensity was determined. Values not sharing a letter are significantly different (*P*<0.05).

To evaluate the preventive effect of nanoparticle curcumin on the development of DSS colitis, mice were treated with nanoparticle curcumin for 7 days prior to the start of DSS administration. As shown in Fig 1A, body weight was significantly lower in the DSS group than the DSS plus nanoparticle curcumin group. The disease activity index was significantly higher in the DSS group than in the DSS plus nanoparticle curcumin group (Fig 1B). Colon length shortening was much more severe in the DSS group than the DSS plus nanoparticle curcumin group (Fig 1C). Colon weight/length ratio, a marker of tissue edema, was significantly higher in the DSS group than in the DSS plus nanoparticle curcumin group (Fig 1D). The histological inflammatory score was significantly lower in the DSS plus nanoparticle curcumin group than in the DSS group (Fig 2A and 2B). Furthermore, epithelial permeability was assessed using FITC-labeled dextran. Serum levels of orally administered FITC-dextran were significantly elevated in the DSS group (Fig 2C), but this elevation was significantly reduced in the DSS plus nanoparticle curcumin group (Fig 2C). These observations indicate that treatment of nanoparticle curcumin suppressed the development of DSS colitis.

### Nanoparticle curcumin suppresses the activation of NF-κB in the colonic epithelial cells

**Fig 3:**
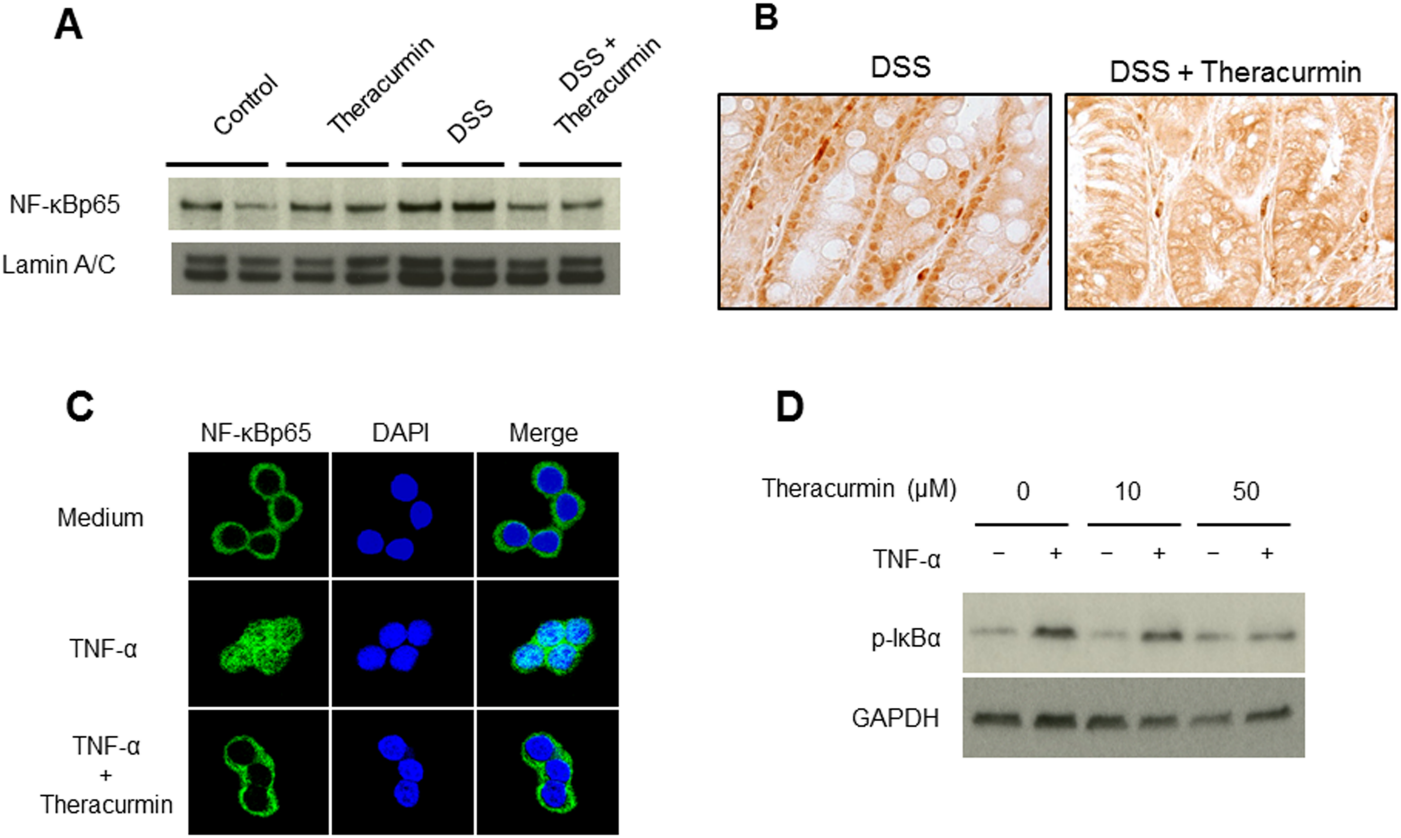
The effect of nanoparticle curcumin on NF-κB activation. (A) Immunoblot for NF-κBp65 in the nuclear protein of colonic epithelium. Lamin A/C was used as a loading control. The picture is representative of four independent experiments. (B) Immunohistochemical staining for NF-κBp65 in the tissues. (original magnification ×200). NF-κBp65 was detected in the nucleus of the epithelial cells in the DSS group, but this was completely blocked in the DSS plus nanoparticle curcumin group. (C) Immunostaining of NF-κBp65 in HT-29 cells. HT-29 cells were stimulated with TNF-α (100ng/ml) in the presence or absence of nanoparticle curcumin (10μM) for 15 minutes. NF-κB p65, green fluorescence; nucleus, DAPI (blue). (D) The effect of nanoparticle curcumin on IκBα phosphorylation in response to TNF-α. HT-29 cells were stimulated with TNF-α (100 ng/ml) in the presence or absence of nanoparticle curcumin (0μM, 10μM, or 50μM) for 15 minutes, and then lysed with lysis buffer. Lysates were subjected to immunoblot analysis. GAPDH were used as loading control. The data represent four independent experiments.

It has been reported that curcumin suppresses the activation of transcription factor NF-κB, which regulates the expression of a number of inflammatory genes [24, 25]. Nuclear protein was extracted from colonic tissues and subjected to immunoblotting. As shown in Fig 3A and S1 Fig. A, translocation of NF-κBp65 into the nucleus was markedly suppressed in the DSS plus nanoparticle curcumin group compared to that of the DSS group. Activation of NF-κB was also detected by immunohistochemical staining in the tissues (Fig 3B). NF-κBp65 was detected in the nucleus of the epithelial cells of the DSS group, but this was completely blocked in the DSS plus nanoparticle curcumin group. These findings indicate that nanoparticle curcumin suppresses the activation of NF-κB in the colonic epithelial cells.

To investigate the direct effect of nanoparticle curcumin on colonic epithelial cells, we used HT-29 cells. As shown in Fig 3C, immunohistochemical analysis showed that NF-κB p65 translocated into the nucleus as early as 15 min. in response to TNF-α (100ng/ml). On the other hand, this response was markedly suppressed by the treatment with nanoparticle curcumin. Similarly, immunoblotting analysis indicated that the phosphorylation of IκBα, which is required for the activation of NF-κB [18], was markedly suppressed in the cells stimulated by TNF-α plus nanoparticle curcumin as compared to the cells stimulated by TNF-α in dose-dependent manner (Fig 3D and S1B Fig).

### Nanoparticle curcumin suppresses the expression of proinflammatory mediators

**Fig 4:**
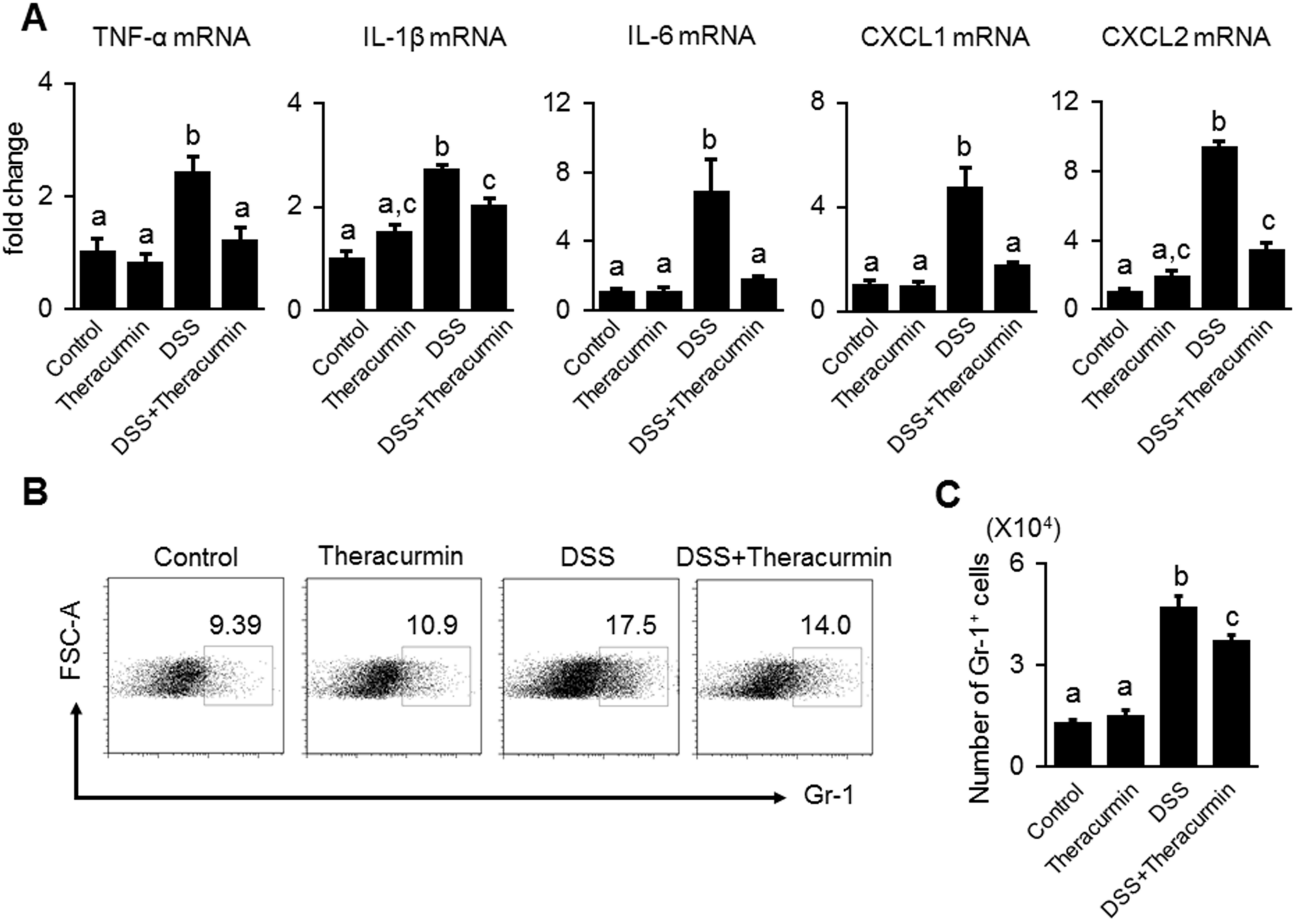
The effect of nanoparticle curcumin on the expression of proinflammatory mediators and neutrophil infiltration. (A) Real-time PCR analysis for the mucosal mRNA expression of TNF-α, IL-1β, IL-6, CXCL1 and CXCL2. The cytokine mRNA expression was converted to a value relative to β-actin mRNA expression, and presented as an increase relative to the control mice. The data are expressed as means ± SEM (n = 6 mice/group). Values not sharing a letter are significantly different (*P*<0.05). (B) Proportion of Gr-1^+^ neutrophils in the lamina propria of the colon. Mucosa Gr-1^+^ neutrophils were analyzed by flow cytometry. (C) The number of Gr-1^+^ neutrophils. The data are expressed as means ± SEM (n = 6 mice/group). Values not sharing a letter are significantly different (*P*<0.05).

Since it is well known that NF-κB mediates the induction of proinflammatory cytokines and chemokines which are involved in the pathogenesis of IBD [18], we next examined whether nanoparticle curcumin suppressed the mucosal mRNA expression of proinflammatory cytokines and chemokines using real-time PCR. As shown in Fig 4A, the treatment of nanoparticle curcumin significantly suppressed mucosal mRNA expression of TNF-α, IL-1β, IL-6, CXCL1 and CXCL2 in colonic epithelial tissues.

Since CXCL1 and CXCL2 are chemokines for neutrophils [26], we evaluated infiltration of Gr-1-positive neutrophils in the colonic mucosa. Flow cytometry analysis showed that the number of Gr-1-positive neutrophils in the colonic mucosa was significantly reduced in the DSS plus nanoparticle curcumin group compared to the DSS group (Fig 4B and 4C). This result indicates that nanoparticle curcumin suppressed the expression of proinflammatory cytokines and chemokines, and consequently reduced the infiltration of neutrophils in the colonic mucosa.

### Effect of nanoparticle curcumin on the gut microbial composition

**Fig 5:**
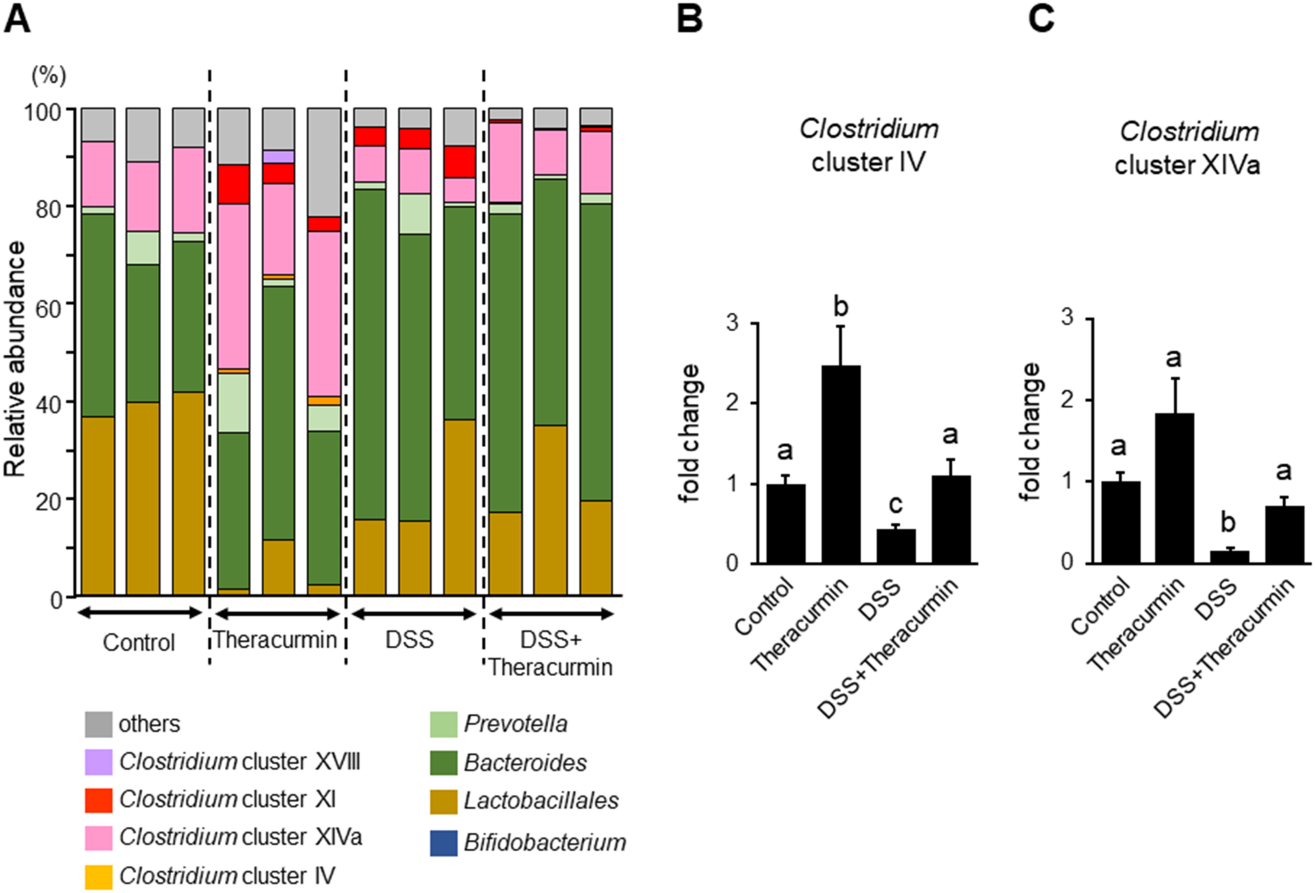
The effect of nanoparticle curcumin on the gut microbial structure. (A) T-RFLP analysis of the gut microbiota. The value indicates the percentage of the predicted bacteria. (B) Real-time PCR analysis for *Clostridium* cluster IV. (C) Real-time PCR analysis for *Clostridium* subcluster XIVa. The values were normalized to the amount of total bacteria, and presented as relative amount to the control group. The data were expressed as means ± SEM (n = 4 mice/group). Values not sharing a letter are significantly different (*P*<0.05).

The previous study reported that polyphenols modulated the gut microbial composition [27]. As curcumin is a type of polyphenol, we investigated the effect of nanoparticle curcumin on the fecal microbial composition using real time-PCR. As shown in Fig 5A and Table 1, T-RFLP analysis predicted that proportion of *Clostridium* clutster IV, *Clostridium* subcluster XIVa, and *Clostridium* cluster XI, significantly increased and that of *Lactobacillales* significantly decreased in the nanoparticle curcumin group as compared to control group. To confirm these findings, we examined the abundance of *Clostridium* cluster IV and *Clostridium* subcluster XIVa using real-time PCR. As shown in Fig 5B and 5C, the abundances of *Clostridium* cluster IV and *Clostridium* subcluster XIVa were significantly decreased in the DSS group as compared to the control group. The abundances of *Clostridium* cluster IV and *Clostridium* subcluster XIVa was significantly increased in the DSS plus nanoparticle curcumin group as compared to the DSS group.

**Table 1:** Effects of nanoparticle curcumin on fecal microbial composition.

Predicted bacteria	Control	Theracurmin	DSS	DSS + Theracurmin
*Bifidobacterium*	0.0	0.0	0.0	0.0
*Lactobacillales*	39.5±2.1^a^	5.3±4.54^b^	22.5±9.7^ab^	24.1±7.9^ab^
*Bacteroides*	33.5±5.8	38.4±9.5	56.6±10.0	57.3±4.9
*Prevotella*	3.3±2.4	6.3±4.3	3.5±3.4	1.6±0.6
*Clostridium*	15.0±1.7^a^	35.8±7.0^b^	12.2±0.9^a^	13.7±3.1^a^
*Clostridium* cluster IV	0.0^a^	1.1±0.3^b^	0.1±0.1^a^	0.2±0.3^a^
*Clostridium* subcluster XIVa	15.0±1.7^a^	28.7±7.1^b^	7.2±1.7^a^	12.8±2.8^a^
*Clostridium* cluster XI	0.0^a^	5.2±2.1^b^	4.9±1.3^b^	0.6±0.3^a^
*Clostridium* subcluster XVIII	0.0	0.8±1.1	0.0	0.1±0.2

The fecal microbial composition was evaluated by T-RFLP method. Each value indicates the percentage of predicted bacteria. Values were expressed as mean ± SEM. Values no sharing a letter are significantly different.

**Fig 6:**
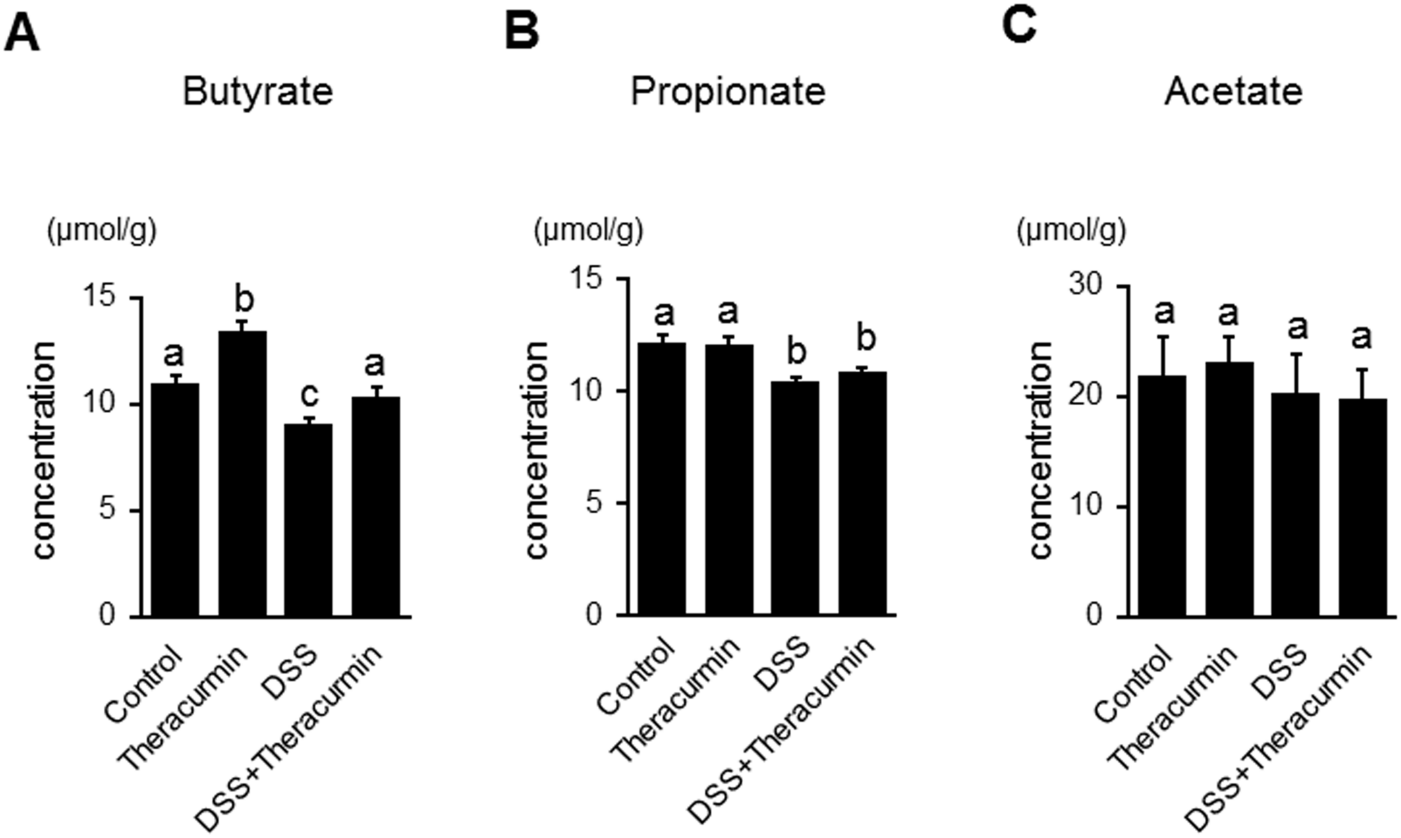
Effect of nanoparticle curcumin on the fecal short-chain fatty acid (SCFA) levels. The concentrations of fecal SCFAs were measured by high-performance liquid chromatography. The data were expressed as means ± SEM (n = 6 mice/group). Values not sharing a letter are significantly different (*P*<0.05).

Short-chain fatty acids (SCFAs) are induced by commensal bacteria during fermentation of dietary fiber [3]. Previous studies reported that *Clostridium* cluster IV and *Clostridium* sub*c*luster XIVa are butyrate-producing bacteria and are associated with induction of regulatory T cells (Tregs) in the colon [28, 29]. As shown in Fig 6A, the fecal butyrate level significantly increased in the nanoparticle curcumin group compared to the control group. Fecal butyrate and propionate levels significantly decreased in the DSS group compared to the control group (Fig 6A and 6B). The fecal butyrate level significantly increased in the DSS plus nanoparticle curcumin group compared to the DSS group. We also measured the fecal acetate levels, but there was no significant difference between the nanoparticle curcumin group and the control group. Similarly, there was no difference between the DSS group and the DSS plus nanoparticle curcumin group (Fig 6C). These findings indicate that nanoparticle curcumin increased the fecal butyrate levels in the inflamed colon as well as in normal colon.

### Nanoparticle curcumin induces CD4^+^ Foxp3^+^ regulatory T cells and CD103^+^ CD8α^−^ CD11c^+^ dendritic cells

**Fig 7:**
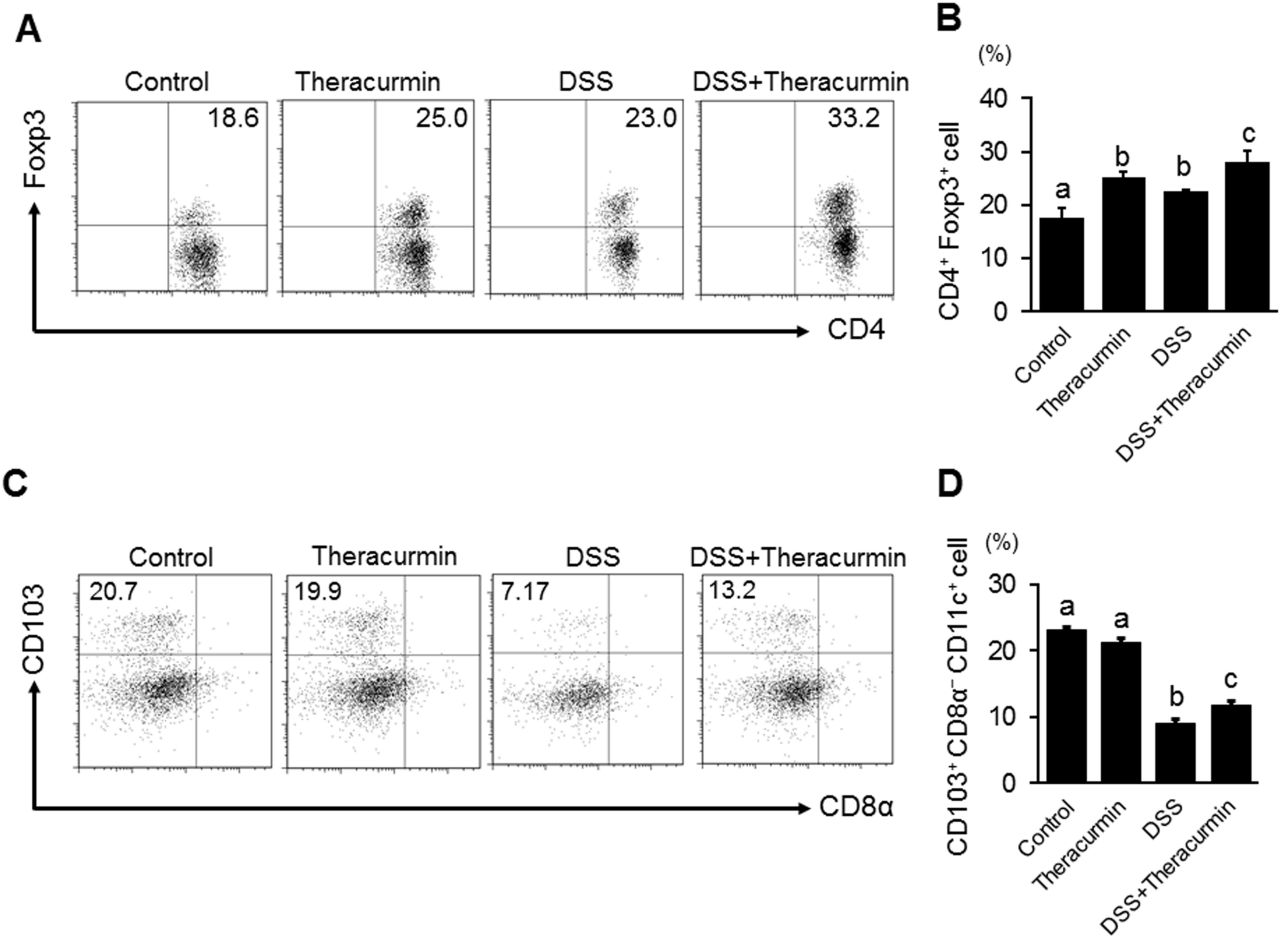
The effects of nanoparticle curcumin on the induction of Tregs and regulatory DCs in the lamina propria of the colon. (A) Flow cytometry analysis for CD4^+^ Foxp3^+^ Tregs in the lamina propria of the colon. Representative picture from two independent experiments. (B) Proportion of CD4^+^ Foxp3^+^ Treg cells in CD4^+^ cells in the lamina propria. The data are expressed as means ± SEM (n = 6 mice/group). Values not sharing a letter are significantly different (*P*<0.05). (C) Flow cytometry analysis for CD103^+^ CD8α^−^ DCs in the lamina propria of the colon. Representative picture from two independent experiments. (D) Proportion of CD103^+^ CD8α^−^ DCs in CD11c^+^ cells in the lamina propria. The data are expressed as means ± SEM (n = 6 mice/group). Values not sharing a letter are significantly different (*P*<0.05).

Recent studies have demonstrated that butyrate plays an important role in the induction of mucosal Tregs [30, 31]. So, we investigated whether nanoparticle curcumin increased Tregs in the colonic mucosa. As shown in Fig 7A and 7B, the proportion of CD4^+^Foxp3^+^ Tregs significantly increased in the DSS plus nanoparticle curcumin group compared to the DSS group. Interestingly, even in normal mucosa, the proportion of Tregs significantly increased in the nanoparticle curcumin group compared to the control group.

Recent studies suggested that CD103^+^ dendritic cells (DCs), especially CD103^+^ CD8α^−^ DCs exert an ability to induce Tregs [32]. Therefore, we investigated whether regulatory DCs were induced by nanoparticle curcumin in the colonic mucosa. Flow cytometry showed that the proportion of CD103^+^ CD8α^−^ DCs significantly decreased in the DSS group compared to the control group (Fig 7C). However, the proportion of these cells was significantly higher in the DSS plus nanoparticle curcumin group than in the DSS group (Fig 7D). These findings suggested that nanoparticle curcumin induced CD103^+^ CD8α^−^ DCs in the inflamed colon.

## Discussion

We demonstrated that nanoparticle curcumin effectively suppressed the development of DSS-induced colitis through both inhibition of NF-κB activation and induction of mucosal Tregs. Treatment with nanoparticle curcumin induced an alteration of gut microbial structure, and some parts of Treg induction might be associated with this microbial change.

Recent reviews reported that 30 to 50% of patients with IBD use complementary and alternative medicine (CAM) [33, 34]. CAM is defined as a group of diverse medical systems, practices and products that are not presently considered to be part of conventional medicine [34]. CAM is usually used by patients who feel an inadequate response to available medications or concerns over side effects. In these reviews, curcumin is described as one of a few established CAM whose clinical effects and safety are validated by certain clinical trials [33, 34]. Curcumin is considered for induction therapy in mild to moderately active UC patients without a response to optimized mesalamine, who do not want dose escalation to immune modulators or biologics [33, 34]. Curcumin can also be used as a supplementary therapy for maintaining remission on optimized mesalamine [33, 34]. Although the clinical usefulness of curcumin has been established in IBD treatment, one of the concerns for oral administration of curcumin was its poor bioavailability associating with its poor water solubility.

Nanoparticle curcumin possesses improved water solubility and oral bioavailability, leading to easy absorption from the gut. Significantly higher elevation of serum curcumin levels after oral nanoparticle curcumin compared to oral curcumin powder have been shown in rats and human [12]. These lead to a possibility of clinical application of nanoparticle curcumin as a CAM for various inflammatory disorders including IBD. In this study, we presented the first basic evidence that nanoparticle curcumin is a potential candidate for a new therapeutic option for IBD.

Suppression of DSS colitis by treatment with nanoparticle curcumin was associated with inhibition of mucosal NF-κB activation. NF-κB is a crucial transcription factor which mediates transcriptional activation of many inflammatory genes [18]. Under the normal physiological state, NF-κB exists in the cytoplasma as a heterodimer complex of p65/p50 subunits. Inflammatory stimuli induce translocation of the NF-κB molecule into the nucleus and active transcription of inflammatory genes. In this study, we showed that the NF-κBp65 subunit was detected in the nucleus of epithelial cells in DSS-treated mice. However, translocation of NF-κBp65 was markedly blocked in the mice treated with DSS plus nanoparticle curcumin. In addition, *in vitro* experiments using HT-29 cells showed that nanoparticle curcumin directly blocked NF-κB activation in intestinal epithelial cells. These results indicate that oral administration of nanoparticle curcumin directly suppressed mucosal inflammation through the inhibition of NF-κB activation.

The gut microbiota and their metabolites play a key role in the pathophysiology of IBD [2, 3]. Although curcumin exerts a wide range of biological effects, there are no reports about its behavior on the gut microbiota. Recent studies have shown that commensal bacteria, such as *Clostridium* cluster IV and XIVa, play an important role in the induction of mucosal Tregs through butyrate generation [31, 35]. Butyrate is one of the short-chain fatty acids which are the major byproducts of fermentation of dietary fiber by commensal bacteria [3]. Tregs are an immune-suppressive subpopulation of helper T cells and generally downregulate induction and proliferation of effector T cells [31, 35]. In this study, we found that treatment with nanoparticle curcumin increased the abundance of high butyrate producing bacteria, *Clostridium* cluster IV and XIVa, and this was accompanied by elevation of fecal butyrate levels. Furthermore, the treatment with nanoparticle curcumin induced an expansion of Tregs in the colonic mucosa. These observations suggested that modulation of the gut microbiota might be a potential mechanism underlying the inhibitory effects of nanoparticle curcumin on DSS colitis.

Recent study demonstrated that DSS treatment induces an expansion of *Bacteroides* species in mice [36]. In particular, *B*. *vulgatus* plays a critical role in the development of colitis through its sialidase activity. In our study, *Bacteroides* tended to increase in DSS-treated mice, but nanoparticle curcumin had no effect on DSS-induced expansion of *Bacteroides*. This indicates that therapeutic effect of nanoparticle curcumin was not associated with modulation of *Bacteroides* expansion.

As mentioned above, curcumin is one of polyphenols. The prebiotic effect of polyphenols has been described using both *in vitro* assays with human gut microbiota, and *in vivo* preclinical studies and clinical trials in which polyphenol and polyphenol-rich foods modulated gut microbiota to enhance the growth of lactobacilli and bifidobacterial [37]. Polyphenols have been reported to shape gut microbiota to favor other specific gut microbial species that can provide health benefits to the host [37]. Some gut microbiota catabolites of polyphenols can also exert ‘prebiotic-like’ activity [37]. Thus, our observations in this study suggest that the modulation of gut microbiota by nanoparticle curcumin may be associated with its direct effects.

DCs act as messengers between the innate and the adaptive immune systems. DCs have been considered as potent stimulators of adaptive immunity, but recent studies have shown a regulatory nature of DCs. Intestinal mucosal DCs, particularly the CD103^+^ subpopulation, has regulatory functions linked to the induction of Tregs [38]. Among CD103^+^ DCs, CD103^+^ CD8α^−^ DCs promote the differentiation of naïve T cells into Tregs by conversion of vitamin A to retinoic acid [32]. In this study, we found that treatment with nanoparticle curcumin enhanced the induction of CD103^+^ CD8α^−^ DCs in the colonic mucosa. Taken together, induction of regulatory DCs by nanoparticle curcumin might contribute to the induction of Tregs in the colonic mucosa.

In conclusion, nanoparticle curcumin suppressed the development of DSS-induced colitis via the suppression of NF-κB activation and the expansion of Tregs. This was accompanied by an alteration of the gut microbial structure and fecal SCFA levels. These findings indicate that nanoparticle curcumin is a novel candidate as a therapeutic option for the treatment of IBD.

## Supporting information

S1 Fig Quantitative values of the result of Immunoblot analysis. (A) The quantitative values of immunoblot analysis for NF-κBp65 presented in Fig 3A was analyzed using ImageJ software (NIH, Bethesda, MD). Data are relative intensity of NF-κBp65 to laminin and expressed as means ± SEM (n = 6). Values not sharing a letter are significantly different (*P*<0.05). (B) The quantitative values of immunoblot analysis of phosphorylated IκBα in Fig 3D was analyzed using ImageJ software. Data are relative intensity of phosphorylated IκBα to GAPDH and expressed as means ± SEM (n = 6). Values not sharing a letter are significantly different (*P*<0.05).
(TIF)

S1 Table Antibodies used in this study. (DOCX)

S2 Table PCR primers used in this study. (DOCX)
