# Supplementary figures and images for: Nanoparticle curcumin ameliorates experimental colitis via modulation of gut microbiota and induction of regulatory T cells
Source: PLoS One. 2017 Oct 6;12(10):e0185999. doi: 10.1371/journal.pone.0185999 (PMC5630155; doi:10.1371/journal.pone.0185999)

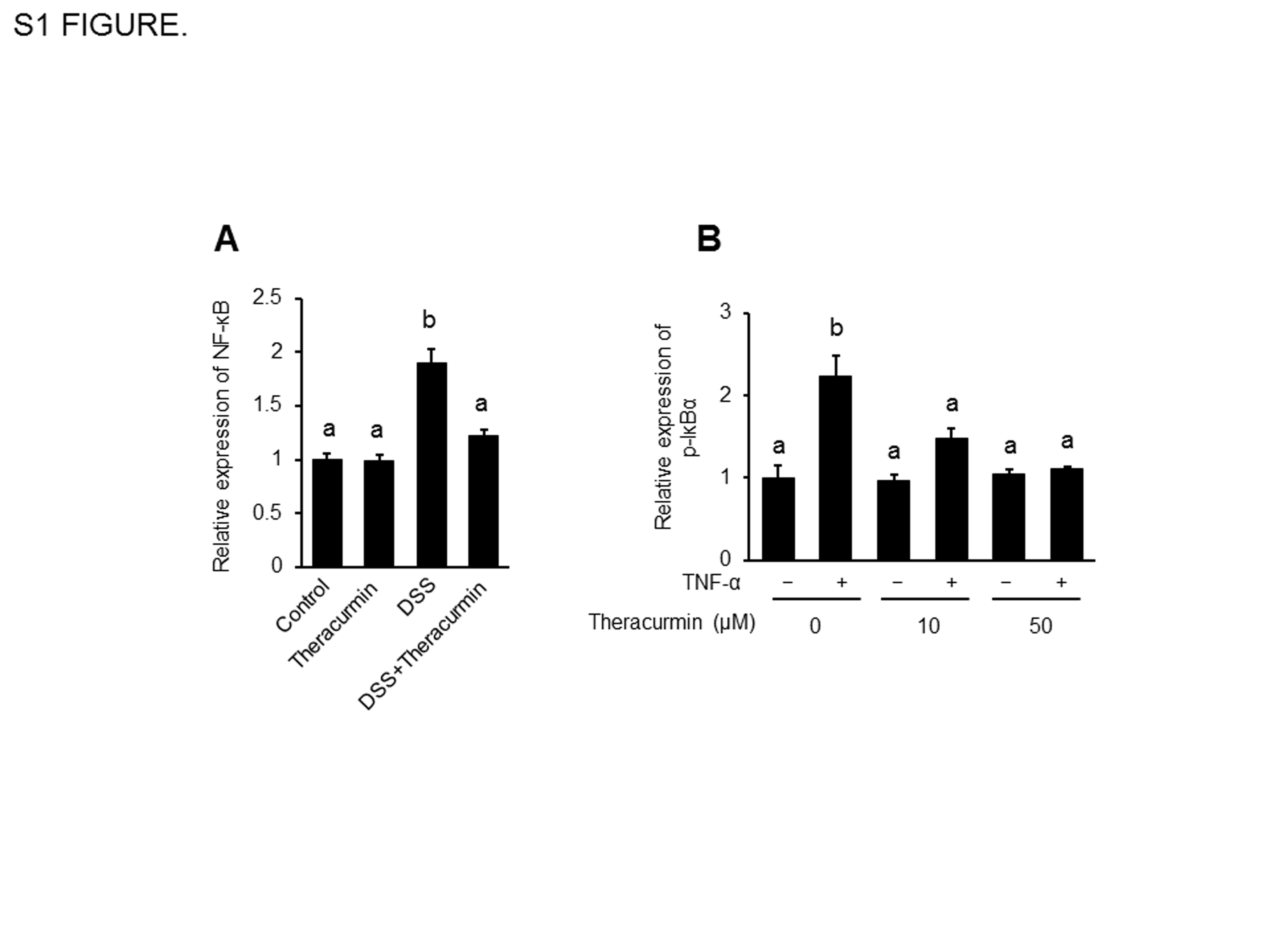

Supplement: S1 Fig — (A) The quantitative values of immunoblot analysis for NF-κBp65 presented in Fig 3A was analyzed using ImageJ software (NIH, Bethesda, MD). Data are relative intensity of NF-κBp65 to laminin and expressed as means ± SEM (n = 6). Values not sharing a letter are significantly different (P<0.05). (B) The quantitative values of immunoblot analysis of phosphorylated IκBα in Fig 3D was analyzed using ImageJ software. Data are relative intensity of phosphorylated IκBα to GAPDH and expressed as means ± SEM (n = 6). Values not sharing a letter are significantly different (P<0.05). (TIF) [file pone.0185999.s001.TIF]
