# Supplementary material for: Nanoparticle curcumin ameliorates experimental colitis via modulation of gut microbiota and induction of regulatory T cells
Source: PLoS One. 2017 Oct 6;12(10):e0185999. doi: 10.1371/journal.pone.0185999 (PMC5630155; doi:10.1371/journal.pone.0185999)
Supplement: S1 Table — (DOCX) [file pone.0185999.s002.docx]

**S1 Table.** Antibodies used in this study

| **Antibodies** | **Clone** | **Manufacturer** |
| --- | --- | --- |
| Alexa Fluor 488-labeled anti-Foxp3 | MF-14 | BioLegend, San Diego, CA |
| FITC-labeled anti- CD11c | N418 | eBioscience, San Diego, CA |
| PE-labeled anti-CD4 | RM4-4 | eBioscience, San Diego, CA |
| PE-labeled anti-CD103 | M290 | BD Biosciences, Franklin Lakes, NJ |
| PE-Cy7-labeled anti-CD8α | 53-6.7 | eBioscience, San Diego, CA |
| APC-labeled anti-Gr-1 | RB6-8C5 | eBioscience, San Diego, CA |
| rabbit anti-NF-κBp65 | C-20 | Santa Cruz Biotechnology, Dallas, TX |
| rabbit phosphorylated-IκBα | 14D4 | Cell Signaling Technology, Beverly, MA |
| rabbit anti-GAPDH | D16H11 | Cell Signaling Technology, Beverly, MA |
| HRP-labeled anti-rabbit IgG |  | GE Healthcare UK Ltd, Little Chalfont, UK |
| Mouse anti-Lamin A/C | 4C11 | Cell Signaling Technology, Beverly, MA |
| HRP-labeled anti-mouse IgG |  | GE Healthcare UK Ltd, Little Chalfont, UK |
| mouse anti-NF-κBp65 | 12H11 | EMD Millipore Corporation, Billerica, MA |
| HRP-labeled anti-mouse IgG |  | Vector Laboratories, Burlingame, CA |

FITC, fluorescein isothiocyanate; PE, phycoerythrin; Cy7, Cyanine7; APC, allophycocyanin; HRP, horseradish peroxidase.
