# Supplementary material for: Nanoparticle curcumin ameliorates experimental colitis via modulation of gut microbiota and induction of regulatory T cells
Source: PLoS One. 2017 Oct 6;12(10):e0185999. doi: 10.1371/journal.pone.0185999 (PMC5630155; doi:10.1371/journal.pone.0185999)
Supplement: S2 Table — (DOCX) [file pone.0185999.s003.docx]

**S2 Table.** PCR primers used in this study

| **Gene, Organism** | **Sequence 5'-3'** | |
| --- | --- | --- |
| IL-1β | sense | CAGGATGAGGACATGAGCACC |
|  | anti-sense | CTCTGCAGACTCAAACTCCAC |
| IL-6 | sense | GACAAAGCCACACTCCTTCAGAGA |
|  | anti-sense | CTAGGTTTGCCGATAGATCTC |
| TNF-α | sense | ATGAGCACAGAAAGCATGATC |
|  | anti-sense | TACAGGCTTGTCACTCGAATT |
| CXCL1 | sense | CCGAAGTCATAGCCACACTC |
|  | anti-sense | CTCCGTTACTTGGGGACACC |
| CXCL2 | sense | AGAAGTCATAGCCACTCTCAAG |
|  | anti-sense | GCTCCTCCTTTCCAGGTCAG |
| β- actin | sense | GTGGGCCGCCCTAGGCACCA |
|  | anti-sense | CGGTTGGCCTTAGGGTTCAGGGGGG |
| Total bacteria | Bact1369F | CGGTGAATACGTTCCCGG |
|  | Bact1492R | TACGGCTACCTTGTTACGACTT |
| *Clostridium* cluster IV | Clep866mF | TTAACACAATAAGTWATCCACCTGG |
|  | Clep1240mR | ACCTTCCTCCGTTTTGTCAAC |
| *Clostridium* cluster XIVa | Ccocc1F | CGGTACCTGACTAAGAAGC |
|  | Ccocc1R | AGTTTYATTCTTGCGAACG |
